# Metabolic and Proteomic Responses to Salinity in Synthetic Nitrifying Communities of *Nitrosomonas* spp. and *Nitrobacter* spp.

**DOI:** 10.3389/fmicb.2018.02914

**Published:** 2018-11-30

**Authors:** Chiara Ilgrande, Baptiste Leroy, Ruddy Wattiez, Siegfried Elias Vlaeminck, Nico Boon, Peter Clauwaert

**Affiliations:** ^1^Center for Microbial Ecology and Technology, Ghent University, Ghent, Belgium; ^2^Department of Proteomics and Microbiology, Research institute for Biosciences, University of Mons, Mons, Belgium; ^3^Research Group of Sustainable Energy, Air and Water Technology, Department of Bioscience Engineering, University of Antwerp, Antwerp, Belgium

**Keywords:** stress response, proteome analysis, carbon metabolism, osmolytes, pure culture, MELiSSA, activity test

## Abstract

Typically, nitrification is a two-stage microbial process and is key in wastewater treatment and nutrient recovery from waste streams. Changes in salinity represent a major stress factor that can trigger response mechanisms, impacting the activity and the physiology of bacteria. Despite its pivotal biotechnological role, little information is available on the specific response of nitrifying bacteria to varying levels of salinity. In this study, synthetic communities of ammonia-oxidizing bacteria (AOB *Nitrosomonas europaea* and/or *Nitrosomonas ureae*) and nitrite-oxidizing bacteria (NOB *Nitrobacter winogradskyi* and/or *Nitrobacter vulgaris*) were tested at 5, 10, and 30 mS cm^-1^ by adding sodium chloride to the mineral medium (0, 40, and 200 mM NaCl, respectively). Ammonia oxidation activity was less affected by salinity than nitrite oxidation. AOB, on their own or in combination with NOB, showed no significant difference in the ammonia oxidation rate among the three conditions. However, *N. winogradskyi* improved the absolute ammonia oxidation rate of both *N. europaea* and *N. ureae*. *N. winogradskyi*’s nitrite oxidation rate decreased to 42% residual activity upon exposure to 30 mS cm^-1^, also showing a similar behavior when tested with *Nitrosomonas* spp. The nitrite oxidation rate of *N. vulgaris*, as a single species, was not affected when adding sodium chloride up to 30 mS cm^-1^, however, its activity was completely inhibited when combined with *Nitrosomonas* spp. in the presence of ammonium/ammonia. The proteomic analysis of a co-culture of *N. europaea* and *N. winogradskyi* revealed the production of osmolytes, regulation of cell permeability and an oxidative stress response in *N. europaea* and an oxidative stress response in *N. winogradskyi*, as a result of increasing the salt concentration from 5 to 30 mS cm^-1^. A specific metabolic response observed in *N. europaea* suggests the role of carbon metabolism in the production of reducing power, possibly to meet the energy demands of the stress response mechanisms, induced by high salinity. For the first time, metabolic modifications and response mechanisms caused by the exposure to salinity were described, serving as a tool toward controllability and predictability of nitrifying systems exposed to salt fluctuations.

## Introduction

Nitrification is a keybioprocess in both natural and engineered systems (Koops and Pommerening-Röser, 2001; Ahn, 2006). This conversion is typically carried out in two steps. Firstly, the oxidation of ammonia/ammonium (NH_3_/NH_4_^+^) into nitrite (NO_2_^-^) is performed by ammonia-oxidizing bacteria (AOB) or archaea (AOA). Subsequently, nitrite oxidation into nitrate (NO_3_^-^) is performed by nitrite-oxidizing bacteria (NOB). More recently, microorganisms capable of performing complete ammonia oxidation (COMplete AMMonia OXidizer, COMAMMOX) were identified (Daims et al., 2015; van Kessel et al., 2015). While understanding the effect of high salinity is interesting from a physiological viewpoint, it also has practical implications for the biotechnological treatment of specific types of wastewater, e.g., industrial wastewater or urine collected from source-separation. These streams present high salt concentrations or fluctuations in salinity (Marickar, 2010) and can lead to a decline in the nitrification rate, incomplete nitrification or even a complete process failure (Yu et al., 2002).

In most cases, the effect of salinity fluctuation is assessed by determining the activity of undefined microbial communities (Jones and Hood, 1980; Rysgaard et al., 1999). Contradictory results have been described, with some reports indicating higher salt tolerance of AOB (Bassin et al., 2012; Pronk et al., 2014), and others indicating NOB as the most tolerant to salt (Moussa et al., 2006; Coppens et al., 2016). These results are expected to be highly affected by the configuration and operational set-up of the bioreactor (Sudarno et al., 2011). Several species of AOB have been described as moderately halotolerant (*Nitrosomonas europaea, N. eutropha, N. halophile*, and *N. mobilis*) or with obligate salt requirements (*N. marina, N. aestuarii, N. cryotolerans, Nitrosococcus oceani*, and *Nitrosococcus halophilus*). NOB obligately halophilic species include *Nitrobacter* *alkalicus, Nitrococcus mobilis, Nitrospina gracilis*, and *Nitrospira marina* (Koops and Pommerening-Röser, 2001). In estuarine sediments, enriched to treat hypersaline waste streams, NOB were washed out, while the AOB population shifted. *N. europaea* became dominant, while *N. mobilis* was affected (Cui et al., 2016).

Only two studies on single model organisms were reported thus far, both of which only evaluated the oxygen consumption in the first 5 min of salt exposure. The AOB *N. europaea* ATCC 19718 showed inhibition at 100 mM NaCl (Hunik et al., 1992) and a mild inhibition of the NOB *Nitrobacter sp.* ATCC 14123 up to 500 mM NaCl (Hunik et al., 1993). Using pure cultures and synthetic microbial communities as model systems, is not only relevant to understand the effect of salinity, the findings also have an impact on nutrient recovery from urine or nitrogen-enriched streams in systems which require a limited number of microorganisms, such as Regenerative Life Support Systems (RLSS) for space application (Clauwaert et al., 2017). In the Micro Ecological Life Support System Alternative (MELiSSA) of the European Space Agency, for instance, a limited number of microorganisms is required to achieve deterministic control and ensuring a high degree of safety (Gòdia et al., 2002).

To our knowledge, the response of nitrifying bacteria to salinity has not been described on a molecular level thus far. Proteomic analysis on nitrifying bacteria have been limited. Few reports describe the proteomic response in pure cultures of AOB, mainly *Nitrosomonas* species, in relation to reactor operation treatment for nutrient-rich streams (Schmidt et al., 2004; Pellitteri-Hahn et al., 2011; Kartal et al., 2012; Yu et al., 2018). Only one proteomic research described the alterations in the proteome of a *Nitrosomonas* species in the presence of *Nitrobacter* *winogradskyi* and heterotrophic strains (Sedlacek et al., 2016), which is complemented by a transcriptome study of the coculture, performed by Perez et al (Pérez et al., 2015). To the authors knowledge, no analysis of a *Nitrobacter* proteome has been reported thus far. Universal response mechanisms identified in bacteria exposed to high salinity, include the regulation of ion transport to preserve the cell turgor (Krämer, 2010), expression of oxidative stress response enzymes (Mager et al., 2000) and osmolyte production (Storz and Hengge, 2010). All are present at a genomic level in both *N. europaea* and *N. winogradskyi* (Chain et al., 2003; Klotz et al., 2006; Starkenburg et al., 2006, 2008), although their actual expression under salt stress has, according to the authors’ knowledge, not been confirmed yet.

Based on this (Hunik et al., 1992, 1993; Chain et al., 2003; Klotz et al., 2006; Starkenburg et al., 2006, 2008), the following mechanisms are hypothesized to occur in response to increased salinity:

1. **Ammonia oxidation will be inhibited at a lower salinity than nitrite oxidation.**
  Rationale: as observed in previous reports.
2. **Oxidative stress response enzymes (dismutases, hydroxyperoxidases and iron regulation enzymes) are predicted to be present.**
  Rationale: these mechanisms are present in both strains, although key enzymes are missing. Most notably, *N. europaea* lacks genes that are involved in the thioredoxin redox couple, glutathione oxidoreductase, and isozymes of dismutases and hydroxyperoxidases. *N. winogradskyi* lacks heme- and non-heme-containing catalases and peroxide-scavenging cytochrome c peroxidase.
3. **Regulation of the cell permeability is expected.**
  Rationale: numerous gene encoding transporters for inorganic ions are present in the genome of *N. europaea* and *N. winogradskyi*. The Open Reading Frames (ORFs) dedicated to transport in each genome amount to 11.5 and 10%, respectively.
4. **Organic osmolytes production pathways are expected to be upregulated.**
  Rationale: both *N. europaea* and *N. winogradskyi* have the genes involved in the production of organic osmolytes, such as betaine, glycine betaine, and sucrose.
5. **Increase in energy production is expected**.
  Rationale: need to fulfill the energetic burden of coping with salinity.

To corroborate the postulated mechanisms, the use of AOB and NOB in a pure culture and in combination cultures (synthetic communities, obtained from mixing pre-cultivated axenic cultures) was chosen as a strategy to exclude the interactions with heterotrophic bacteria.

As test model for nitrification in high nutrient environments, two *Nitrosomonas* strains (*N. europaea* ATCC 19718 and *N. ureae* Nm10) and two *Nitrobacter* strains (*N. winogradskyi* ATCC 25931 and *N. vulgaris* Z) were combined and their ammonia and nitrite oxidation rates were evaluated. The cultures were exposed to the growth medium without additional NaCl and the growth medium was supplemented with 40 mM and 200 mM NaCl, with a final electrical conductivity (EC) of 5, 10, and 30 mS cm^-1^, respectively. The salinities tested were based on EC levels comparable to the ones reported for nitrification systems, treating 10, 20, and 60% real urine (Coppens et al., 2016; De Paepe et al., 2018). The investigation of the short-term salt response mechanisms (5 versus 30 mS cm^-1^) of a *N. europaea* ATCC 19718 and *N. winogradskyi* ATCC 25391 co-culture was achieved through proteomic analysis.

## Materials and Methods

### Strain Selection and Cultivation

*Nitrosomonas europaea* ATCC 19718, *N. ureae* Nm10, *N. winogradskyi* ATCC 25931 and *N. vulgaris* Z originate from the culture collection at the Department of Microbiology and Biotechnology, University of Hamburg, Hamburg, Germany. The strains were axenically cultivated at 28°C in an orbital shaker (KA, KS 4000 i control, Germany). The AOB were grown in an ATCC medium 2265, while the NOB was first grown in a mixotrophic medium (DSMZ medium 756a, 756b), washed three times with PBS and transferred to an autotrophic medium (DSMZ medium 756c) 2 weeks prior the activity tests.

### Activity Tests

In the activity tests, strains and communities had the same initial OD_600_. AOB and NOB communities were prepared mixing equal volumes of AOB and NOB cultures, cultivated as previously described, with equal OD_600_, in a substrate depleted mineral medium containing FeSO_4_ × 7 H_2_O 0.0025 g L^-1^, NaH_2_PO_4_ 0.71 g L^-1^, KH_2_PO_4_ 0.68 g L^-1^, (NH_4_)_6_Mo_7_O_24_ 0.177 g L^-1^, ZnSO_4_ × 7 H_2_O 0.0043 mg L^-1^, CuSO_4_ × 5 H_2_O 0.0041 mg L^-1^, MgSO_4_ 7H_2_O 0.052 g L^-1^, CaCl_2_ 2H_2_O 0.74 mg L^-1^, NaHCO_3_ 0.8 g L^-1^ to a final OD_600_ of 0.1. Pure cultures with OD_600_ of 0.1 were included as controls. Electrical conductivity (EC) measurements were conducted at room temperature (25°C) with a Multi-Channel Analyser (CONSORT C833, Turnhout, Belgium). The medium was adjusted to the desired EC (5, 10, or 30 mS cm^-1^) with the addition of NaCl (0, 40, and 200 mM NaCl, respectively) and the initial pH of the medium was 7.5. The nitrogen concentrations and oxidation rates will be discussed with reference to the total nitrogen values. The synthetic communities with both AOB and NOB were provided with a substrate concentration of 80 mg-N L^-1^ of NH_4_Cl (5.7 mM of NH_4_^+^) and 80 mg-N L^-1^ of NaNO_2_ (5.7 mM of NO_2_^-^) to evaluate the ammonia and nitrite oxidation rates, respectively, while the pure strains were fed only with 80 mg-N L^-1^ of NH_4_Cl for the AOB and 80 mg-N L^-1^ of NaNO_2_ for the NOB. All the activity tests were performed in triplicate, as reported by Lindeboom et al. (2018). For each culture, 250 μL duplicates were prepared in a sterile flat-bottom 96-well plate and incubated at 28°C in the dark, in a Thermoshaker (MB100-4A, Thermoshakers Aosheng, China) for up to 6 days or until complete consumption of the substrate. An orbital shaking of 600 rpm was applied to prevent oxygen limitation. Ammonium and nitrite concentrations were spectrophotometrically monitored daily for each culture, with the exception of the pure NOB culture, which was exposed only to nitrite. The ammonium concentration was determined by the Berthelot reaction (Bucur et al., 2006) and the nitrite concentration was determined by the Montgomery reaction (Montgomery and Dymock, 1961). Technical triplicates were utilized for each analysis. All spectrophotometric measurements were performed using a Microplate Readers Infinite^®^F50 (Tecan Group Ltd., Germany). An overview of the consortia and their main characteristics are presented in Table 1. All the results are expressed as mean ± SD. Since the preparation of the synthetic communities resulted in a halved concentration of AOB or NOB cells in the mixes, compared to the pure cultures, the rates have been normalized accordingly. A Mann-Whitney *U*-test method and One Sample *Z*-test was applied for statistical analysis. The level of significance was established at *p* ≤ 0.05.

**Table 1:** Overview of the individual strains and of the composition of the nitrifying synthetic communities.

		**Ammonia-oxidizing bacteria**		**Nitrite-oxidizing bacteria**	
		*Nitrosomonas europaea* ATCC 19718	*Nitrosomonas ureae* Nm10	*Nitrobacter winogradskyi* ATCC 25391	*Nitrobacter vulgaris* Z

**Communities composition**	E	X			
	U		X		
	W			X	
	V				X
	EW	X		X	
	UW	X			X
	EV		X	X	
	UV		X		X
	EUWV	X	X	X	X

### Proteomic Analysis

The *N. europaea* ATCC 19718 and *N. winogradskyi* ATCC 25391 consortium was selected for the proteomic analysis. The strains were cultivated independently as previously described. The synthetic communities were assembled, mixing equal volumes of the *N. europaea* ATCC 19718 and *N. winogradskyi* ATCC 25391 cultures with the same OD_600_ (Montràs et al., 2008; Cruvellier et al., 2016) and transferred in a media containing FeSO_4_ × 7 H_2_O 0.0025 g L^-1^, NaH_2_PO_4_ 0.71 g L^-1^, KH_2_PO_4_ 0.68 g L^-1^, (NH4)_6_Mo_7_O_24_ 0.177 g L^-1^, ZnSO_4_ × 7 H_2_O 0.0043 mg L^-1^, CuSO_4_ × 5 H_2_O 0.0041 mg L^-1^, MgSO_4_ 7H_2_O 0.052 g L^-1^, CaCl_2_ 2H_2_O 0.74 mg L^-1^, NaHCO_3_ 0.8 g L^-1^ and (NH_4_)_2_SO_4_ 1.32 g L^-1^. The co-culture was incubated in replicates (*n* = 6) in the same conditions as the pure strains. A twofold subculture was performed weekly for 4 weeks, after which the synthetic communities were inoculated in fresh media with either 5 or 30 mS cm^-1^ EC, adjusted with the addition of NaCl (Supplementary Figure S3). No nitrite accumulated neither at 5 nor at 30 mS cm^-1^ (Supplementary Figure S3). The availability of nitrite, originating from the ammonia oxidation of *N. europaea*, limited the growth of *N. winogradskyi* in both conditions. After 7 days of exposure to salinity, the pellet was collected and the proteins extracted as described previously (Meur et al., 2018). Briefly, proteins were extracted in guanidinium chloride 6 M using ultrasonication. After reduction and alkylation, proteins were precipitated with cold acetone and digested with trypsin at an enzyme substrate ratio of 1:50. Peptides were analyzed using a label free quantitative workflow as described by Leroy and collaborators (Leroy et al., 2015). Data were then acquired on a TripleT of 5600 mass spectrometer (SCIEX, Benelux) coupled online with an Eksigent 2D ultra LC (SCIEX, Benelux) equipped with a 25 cm C18 column. After a MS spectrum (m/z range 400–1200), 50 MS/MS were acquired at each cycle in a m/z range of 100 to 1800, with an accumulation time of 50 ms. The total duty cycle was kept below 3 sec to ensure accurate quantification. A database search was performed using ProteinPilot 4.5 (SCIEX, Benelux) using all UniProt entries for *N. europaea* and *N. winogradskyi* (release July 2016). A False Discover Rate (FDR) was estimated using a revert database strategy and only proteins below the threshold of 1% of the FDR were further considered. For quantification, the PeakView version 1.1.1 was used to generate extracted ion chromatograms (XIC) and only proteins quantified with 2 or more peptides were further analyzed. The EggNOG 4.5.1 functional classifier (Huerta-Cepas et al., 2015) was utilized for the assignment of Clusters of Orthologous Groups (COGs).

## Results

### Activity Measurement

#### Ammonia Oxidation

**FIGURE 1:**
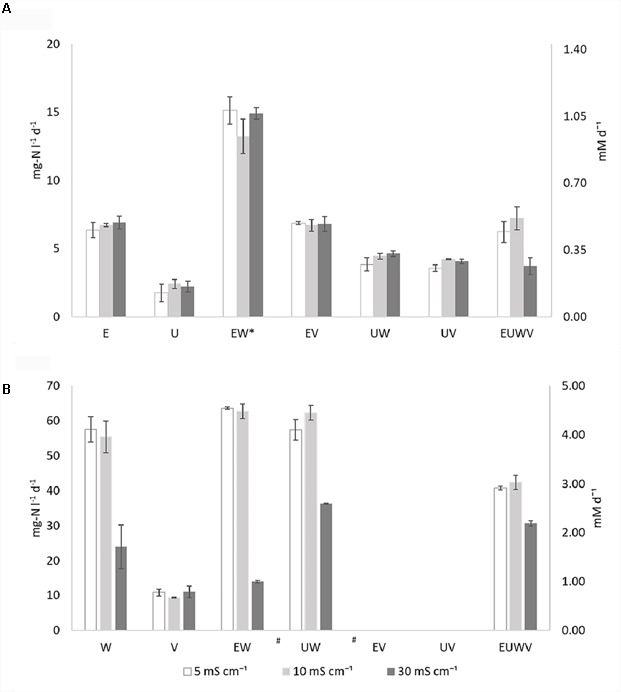
Overview of the ammonia oxidation rates **(A)** and nitrite oxidation rates **(B)** expressed as mg-N l^-1^ d^-1^ and mM d^-1^ (secondary axis) at different salinities (EC: 5, 10, and 30 mS cm^-1^) for the individual strains *N. europaea* (E), *N. ureae* (U), *N. winogradskyi* (W), *N. vulgaris* (V) and synthetic communities combining *N. europaea* and *N. winogradskyi* (EW), *N. ureae* and *N. winogradskyi* (UW), *N. europaea* and *N. vulgaris* (EV), *N. ureae* and *N. vulgaris* (EV), *N. europaea, N. ureae, N. winogradskyi*, and *N. vulgaris* (EUWV) (*n* = 3) at OD_600_ of 0.1. The communities marked with (^∗^) present an activity which is significantly different than the others (*p* < 0.05), while the communities marked with (^#^) present a significant difference at 30 mS cm^-1^ (*p* < 0.05).

An overview of the ammonia oxidation rates are presented in Figure 1A, Supplementary Table S1, and Supplementary Figure S1. Ammonia oxidation was observed in all of the AOB strains and all of the synthetic communities were tested (Figure 1A). *N. europaea* (E) rates were, on average, three times higher than *N. ureae* (U) rates, and neither were affected by the salinities tested. No effect from the increased salinity was observed for the synthetic communities with *N. europaea* (EW, EV). The combination EW showed an ammonia oxidation rate that was significantly (*p* < 0.05) higher than the other synthetic communities, for all the conditions tested, and doubled compared to the pure culture. Similarly, the synthetic communities with *N. ureae*, UW and UV, were not affected by salinity. Although the extent of such interaction was not statistically significant (*p* > 0.05), a positive effect of NOB, on the ammonia oxidation activity of U was also observed, without strain specificity. Only the combination of EUWV was sensitive to the higher conductivity, although not significant (*p* > 0.05). At 30 mS cm^-1^ the ammonia oxidation rate measured was 1.9 and 1.7 times lower than at 10 and 5 mS cm^-1^, respectively.

#### Nitrite Oxidation

An overview of the nitrite oxidation rates is presented in Figure 1B, Supplementary Table S1, and Supplementary Figure S2. Nitrite oxidation activity could be observed for the two pure NOB strains separately and for all the synthetic communities without a lag phase, except for EV and UV (Figure 1B). *N. winogradskyi* (W) presented rates six times higher than *N. vulgaris* (V) at 5 and 10 mS cm^-1^ and two times higher at 30 mS cm^-1^. The pure culture of *N. winogradskyi* and its synthetic communities (W, EW, UW) showed a significant (*p* < 0.05) decrease of activity at 30 mS cm^-1^, ranging from 1.6-fold for UW to a 4.5-fold for EW. Neither the presence of *N. europaea* nor *N. ureae* contributed significantly to the rates (*p* > 0.5).

The pure culture of *N. vulgaris* (V) displayed nitrite oxidation activity at a similar rate in all different conditions. The negative or null rates observed for EV and UV, the synthetic communities containing *N. vulgaris* as sole NOB, indicate that the substrate is not being oxidized, and that *N. vulgaris* is therefore not active, even in the absence of additional salts (5 mS cm^-1^). EUWV, the consortium with all the bacteria, presented a similar behavior, with a 1.3 and 1.4-fold decrease among 30 and 10 or 5 mS cm^-1^ (*p* > 0.5).

### Proteomic Analysis

#### Global Overview

**FIGURE 2:**
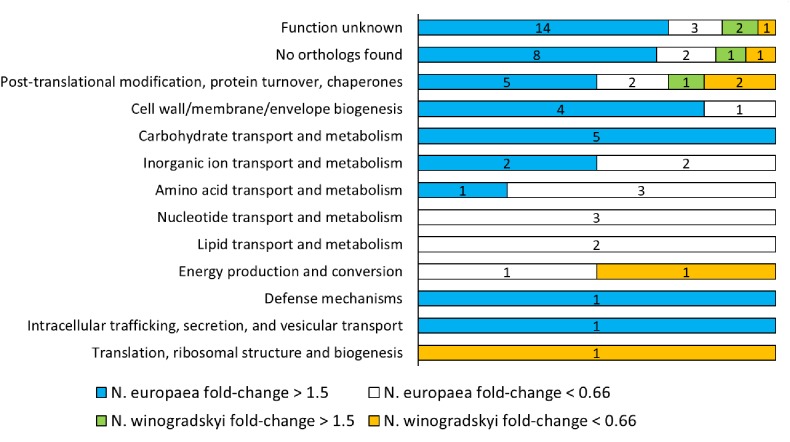
Clusters of Orthologous Groups (COGs) classification of *N. europaea* and *N. winogradskyi* proteins with a significant fold change (higher than 1.5 or lower than 0.66; *p* < 0.05) after exposure to 30 mS cm^-1^.

The combination of *N. europaea* and *N. winogradskyi* (EW) was selected for further characterization, since a synergistic effect for *N. europaea*, resulting in higher rates, was observed (Figure 1) and the full genome sequence is available for the individual strains. A total of 869 proteins were detected, of which 144 belonged to *N. winogradskyi* and 725 to *N. europaea* (Supplementary Table S2). Of these, 70 presented a significant fold-change (higher than 1.5 or lower than 0.66; *p* < 0.05) when exposed to an environment of 30 mS cm^-1^. A lower protein extraction efficiency was observed for *Nitrobacter* and is most likely responsible for the low proportion of *Nitrobacter* proteins. The median of the fold change of *N. europaea*’s proteins was 1.045, while for *N. winogradskyi*’s proteins it was 0.92. This indicated a similar relative abundance of the two strains among samples. An overview of the functional classification is presented in Supplementary Table S3 and Figure 2. Almost half of the proteins detected were either not assigned to any COG, or their function is reportedly unknown. The COG assignment of the remaining 38 proteins suggested that main processes perturbed by salinity exposure, include proteins involved in protein folding and maturation, cell wall biogenesis, carbohydrates and amino acids metabolism, and ion transport (Figure 2).

#### Oxidative Stress in *N. europaea* and *N. winogradskyi*

In addition to an osmotic stress response, cells exposed to 30 mS cm^-1^ presented a higher abundance of proteins typically involved in an oxidative stress response.

In *N. europaea*, the main mechanisms observed, relate to the correction of oxidative damage on disulfide bridges. A principle difference in oxidative-stress related proteins, was observed in the disulfide bond formation (Dsb) system, responsible for the oxidative folding in the periplasmic space. The proteins DsbA (Q82XB9) and DsbC (Q82UH5) were 1.5 and three times more abundant, respectively. Although not significant (*p* = 0.06), DsbE (Q82WC3) was also 1.7 times more abundant. A twofold increase of the periplasmic chaperone SurA (Q82W17) was also observed, as well as a 2.7-fold increase of methionine sulfoxide reductase MsrA (Q82U12).

*Nitrobacter winogradskyi* cells exposed to 30 mS cm^-1^ presented differences mainly in the enzymes involved in the direct inactivation of ROS. Specifically, two alkyl hydroperoxide reductase were found to be 2.3 (Q3SSM2) and 1.7 (Q3SP52) times more abundant.

#### Cell Permeability in *N. europaea*

Significant differences at 30 mS cm^-1^ were observed for mechanisms involved in the preservation of the cell turgor in *N. europaea*. Several transporters were also significantly different. Proteins with a higher abundance were identified as putative a cation-efflux system signal peptide protein (Q82T81, 3.0 times higher), a small metal binding protein SmbP (A0A136MUM4, 2.2 times higher), the HlyD family efflux pump subunit (Q82VH5, 2.0 times higher), and an uncharacterized protein family UPF0003 (Q82TE6, 1.7 times higher). Proteins with a lower abundance were identified as a iron-regulated ABC transporter ATPase subunit SufC (Q82UN3, 1.6 times lower), a TonB-dependent receptor protein (Q82WN2, 1.8 times lower) and an acriflavin resistance protein:Heavy metal efflux pump CzcA (Q820R6, 1.9 times lower).

In addition to the regulation of the transmembrane transport systems, additional mechanisms related to the cell permeability are altered at 30 mS cm^-1^. Among the outer membrane proteins, the most significant alterations at 30 mS cm^-1^ relate to a disparity in the Tol/Pal complex, with Tol periplasmic component (Q82VZ7) being four times more abundant and Pal (Q82XN8) being halved. Additionally, the enzyme involved in the later stage of the terpene biosynthesis pathway, isoprenyl transferase (Q82TZ9), was found to be almost halved at 30 mS cm^-1^. Similarly, polyprenyl synthetase (Q82TH2) was also significantly different (*p* = 0.02), however, the fold change did not reach the set standard (0.8-fold decrease).

### Osmolytes in *N. europaea*

Several proteins potentially involved in the production of osmolytes were significantly different in *N. europaea* cells exposed to 30 mS cm^-1^. A sucrose, synthase (Q820M5), was 2.7 times more abundant, while a PfkB family of carbohydrate kinase (A0A136N5Q5) was almost halved. The pyridoxal phosphate-containing glycine decarboxylase GcvPS (Q82WQ4), the aminomethyltransferase GcvT (A0A136N5F3) and the carrier protein GcvH (A0A136N5F2) were all 1.6 time less abundant at higher salinity. Ultimately, the glucans biosynthesis protein OpgG (Q82SA9) was found to be twofold higher.

#### Energy Production in *N. europaea*

The proteome of cells exposed to 30 mS cm^-1^, did not present a major modification to the enzymes involved in the energy production through nitrogen metabolism. In *N. europaea*, only an ammonia monooxygenase subunit B (Q04508) was found to be almost halved.

**FIGURE 3:**
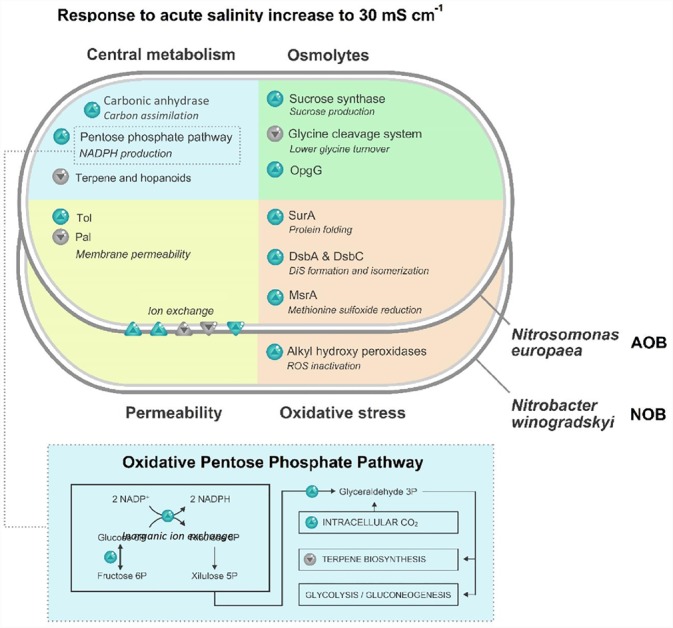
Schematic representation of statistically significant modifications (higher than 1.5 or lower than 0.66; *p* < 0.05) in the proteome of the ammonia-oxidizing bacterium (AOB) *N. europaea* and nitrite-oxidizing bacterium (NOB) *N. winogradskyi* after exposure to 30 mS cm^-1^. Blue arrows indicate a higher abundance of the protein, gray arrows a lower abundance.

Significant differences concerning the carbon metabolism were observed in *N. europaea* cells exposed to 30 mS cm^-1^ (Figure 3). A 1.5-fold increase in carbonic anhydrases (Q82TG2) was detected. Additionally, a higher abundance of enzymes involved in the oxidative phase of the Pentose Phosphate Pathway (PPP) was identified. Glucose-6P isomerase (Q82SP4), glucose-6P 1-dehydrogenase (Q82X90) and 6-phosphogluconate dehydrogenase (Q82X91) were y 1.7, 1.7 and two times more abundant, respectively. Another difference of the PPP, is a twofold increase of a probable phosphoketolase (Q82T07), the enzyme responsible for the conversion of xylulose-5P into glyceraldehyde-3P. A halved abundance of DXP reductoisomerase (A0A136NUH9), the committing enzymes of terpene biosynthesis, was also observed.

## Discussion

### Ammonia Oxidation Is Less Inhibited by Salinity Than Nitrite Oxidation

The results of the activity tests showed that *N. europaea, N. ureae* and *N. vulgaris* are tolerant to the salinities tested. While *N. europaea* was described as tolerant to salinity of up to 400 mM NaCl (∼50 mS cm^-1^), while *Ns urea* has a maximum tolerance of 200 mM NaCl (which corresponds to 25 mS cm^-1^) (Koops et al., 2006). The short length of the exposure is most likely responsible for this apparent discrepancy. *N. winogradskyi* is the only strain that displayed a low tolerance for an environment with 30 mS cm^-1^, yet the observed nitrite oxidation rate was the highest among all tested strains. The comparison between the pure strains and the synthetic communities, with two components, clearly showed that the presence of an additional strain does not modify the response to salinity.

Generally, in reported literature for both AOB and NOB, rates observed are lower than in others (Nowka et al., 2015; Mellbye et al., 2018). This is probably linked to the experimental set up, which differs among studies. Overall, a beneficial contribution of NOB on AOB activity was observed. A similar observation was previously reported by Pérez et al., who identified a lower transcription of the stress-response gene in a *N. europaea* and *N. winogradskyi* co-culture, for both strains (Pérez et al., 2015). Additionally, NOB establish the removal of potentially harmful concentrations of nitrite (Yu and Chandran, 2010), although thresholds reported by Beaumont et al. (2004) and Yu and Chandran (2010) for *N. europaea* (60 mM NaNO_2_ activate denitrification mechanisms in *N. europaea*) were higher than those used in this study. The EV culture represents an exception to this observation, as *N. vulgaris* had no positive effect on the activity of *N. europaea.* Furthermore, the synthetic communities combining *N. vulgaris* with *N. europaea* or *N. ureae* showed a complete inhibition of nitrite oxidation.

Little is known about the physiological characteristics of *N. vulgaris*, so the observed inhibition could be caused by either the presence of AOB, or ammoniacal nitrogen, or a combination of both. Although some reports indicate that the co-occurrence of *Nitrosomonas* spp and *N. vulgaris* is not mandatory for efficient reactor operations (Park et al., 2016), their co-presence was described in mixed communities exposed to 50–60 mg NH_4_^+^-N L^-1^ (Wang et al., 2012). On the other hand, inhibition from ammoniacal nitrogen has been frequently described as a challenge for nitrite oxidation (Anthonisen et al., 1976). While ammonium is not commonly reported as negative for NOB (Ma et al., 2014) and while it did not inhibit *N. winogradskyi* (Figure 1B), free ammonia has been considered a major inhibitor of nitrite oxidation. This effect was reported on a broad range of concentrations, as low as 1 mg NH_3_-N L^-1^ (Anthonisen et al., 1976), which could have been present in this set up (80 mg NH_4_^+^ L^-1^, pH 7.5, temperature 28°C, resulting in 2.3 mg NH_3_-N L^-1^). However, the degree and role of free ammonia on *Nitrobacter* spp. inhibition has recently been questioned, with pH being designated as the primary cause of the inhibition (Hawkins et al., 2010). Nonetheless, it was shown that the transcriptome of *N. winogradskyi* Nb255 was altered in the presence of 25 mM of NH_4_^+^, with notable changes in genes related to nitrogen and carbon assimilation, biofilm/motility, post-translational modification, protein turnover, biogenesis and chaperons (Sayavedra-Soto et al., 2015). Specific studies dedicated to the inhibitory effect of free ammonia on NOB pure cultures are necessary to individuate the cause and extent of this loss of activity. Conversely, AOB in combination with ammoniacal nitrogen did not affect the behavior of *N. winogradskyi* (Figure 1B).

Remarkably, the combination including all the strains tested (EUWV) is the only consortia whose ammonia oxidation is affected by salinity. Perhaps, the competition between two different AOB strains, resulted in an increased vulnerability toward salt stress, however, no proof for this hypothesis was generated. These results demonstrate that even microbial communities with a relatively low complexity can display a different behavior toward an environmental stress, such as salinity. Our work demonstrates that the use of synthetic communities can be a promising tool to study complex microbial interactions.

Overall, the results of the activity tests are in contrast with the two studies performed on pure cultures of *N. europaea* (Hunik et al., 1992) and *N. winogradskyi* (Hunik et al., 1993) that suggested a lower tolerance for salinity in *N. europaea* than in *N. winogradskyi*. *N. europaea* did not show inhibition to a NaCl concentration twice the amount reported and *N. winogradskyi* displayed lower activity at a halved NaCl concentration. Therefore, our hypothesis that salinity has a greater effect on ammonia oxidation than nitrite oxidation was contradicted. This indicates that differences in sample composition, preparation, harvesting and environmental conditions can play a significant role in the preservation of the activity. In particular, the centrifugation and refrigeration performed by Hunik et al. as well as the shorter experiment time, could have impacted the observation to a great extent.

### Regulation of Cell Permeability Related Enzymes in *N. europaea* at 30 mS cm^-1^: Ion Exchange and Tol/Pal System

Several proteins associated to inorganic ion exchange (Protein ID: Q82TE6, Q82T81, Q82VH5, Q820R6, A0A136MUM4, Q82WN2, Q82UN3) were found to be significantly different at 30 mS cm^-1^, indicating that the *N. europaea* deploys a different regulation of the ion and solutes exchange with its environment as a response to a sudden increase in salt concentration.

Interestingly, enzymes associated with membrane permeability other than ion transporters were also found to be different in cells exposed to a higher salinity. The role of the alterations observed in the Tol/Pal system could not be fully determined, as its function is still largely unknown. Nonetheless, mutations in Tol–Pal genes in *E. coli* caused hypersensitivity to chemical stress and disruption of the cell wall (Godlewska et al., 2009). The lower abundance of the two enzymes of the final phase of terpene biosynthesis may be indicative of a metabolic redirection toward the production of hopanoids. These molecules were previously observed in *N. europaea* (Seemann et al., 1999; Sakata et al., 2008) and are thought to function as a stabilizer of the membrane fluidity, which can prevent the diffusions of small molecules and confer resistance to salinity (Sáenz et al., 2012).

Generally, the evaluation of the mechanisms to preserve the cell turgor indicates that *N. europaea* not only regulates the transmembrane transport, but might present modification in the permeability of the cell, due to the different expression of structure related enzymes.

### Oxidative Stress Enzymes in *N. europaea* and in *N. winogradskyi* at 30 mS cm^-1^: Sulfur Oxidation Systems and Hydroperoxide

Oxidative stress is commonly associated with an increase in reactive oxygen species (ROS) that can damage the cell. Proteins are sensitive to oxidative modifications particularly the residues containing disulfide bonds, and a common mechanism to contrast the oxidative stress, is to restore the correct folding of the protein or directly inactivate ROS (Català et al., 2000). Observations of the stress response revealed that, despite the different targets and modes of action, oxidative stress and an osmotic stress response can be simultaneously present. Besides alterations in the above-mentioned proteins, typically associated with an osmotic stress response, oxidative stress response enzymes were also detected in *N. europaea*. At higher salinities, protein upregulation concerned the protein MsrA, which was described as responsible for reducing free and protein-bound methionine-S-sulfoxide, to methionine (Weissbach et al., 2002) and the two effectors (DsbA and DsbC) of the disulfide bond formation Dsb system. These enzymes are, respectively, responsible for introducing new diS-bonds or isomerizing existing ones (Ito and Inaba, 2008). Alterations related to this system can also be found in interacting proteins, such as SurA. This protein in *E. coli*, was shown to interact with both DsbA and DsbC, and with its deletion induced chemical sensitivity (Lazar and Kolter, 1996).

In *N. winogradskyi*, significant differences were observed only in enzymes associated with an oxidative stress response. In *E. coli* Such proteins have been described as efficient scavengers of hydrogen peroxide, a strong growth inhibitor that can damage proteins presenting sulfur residues through thiol oxidation as well as generate radicals (Seaver and Imlay, 2001). The higher abundance of alkyl hydroperoxide reductases at higher salinity are not an exclusive feature of *N. winogradskyi*, but have been previously reported in association with the osmotic stress response (Armstrong-Buisseret et al., 1995). The low resolution on the proteome of *N. winogradskyi* is probably caused by the lower presence of the NOB in the co-culture. While the theoretical AOB/NOB autotrophic ratio is 2, derived by the AOB requirement of two electrons for ammonium activation, that prevent their utilization by the NOB for biomass production (Hagopian and Riley, 1998; Blackburne et al., 2007a,b; Winkler et al., 2012), the maintenance processes (energy consuming processes required for cell survival activities other than formation of biomass), may be affected by several conditions, such as culture conditions and stress (Cruvellier et al., 2016). Maintenance is also linked to thermodynamic efficiency of the energy generating reactions and NOB requires even more energy to generate NAD(P)H through reverse electron flow, due to the lower redox potential of nitrite (Poughon et al., 2001). The work of Pérez et al. (2015) showed that in a chemostat the ratio was 4:1. Therefore, NOB biomass yield is expected to be two times lower than AOB, and can be further reduced by exposure to salinity. Enhanced results could be obtained by optimizing the protein extraction procedures or by growing the cells in bigger volumes or in a more controlled environment (i.e., chemostat).

### Organic Osmolytes Production Enzymes Altered in *N. europaea* at 30 mS cm^-1^: Sucrose Synthase, Glycine Cleavage System and Osmoregulated Periplasmic Glucans

Several differences at 30 versus 5 mS cm^-1^ were observed in crucial enzymes associated with osmolytes production in *N. europaea.* The major modification that was observed was related to two enzymes, associated with sucrose production, one of the most commonly described osmolytes (Empadinhas and da Costa, 2008). Encoded by the same operon, these enzymes are thought to provide a timely sucrose production in response to environmental conditions (Diricks et al., 2015). The glycine cleavage system, involved in the oxidative cleavage of glycine into CO_2_, NH_4_^+^ and a methylene, showed a lower abundance at 30 mS cm^-1^. A lower presence of protein involved in glycine degradation suggests an intracellular need for this amino acid, potentially as a building block for glycine-betaine production, another universal osmolytes (Empadinhas and da Costa, 2008). However, no enzymes belonging to its biosynthesis or transport were identified.

An unexpected result was the higher abundance of an enzyme required for the branching of Osmoregulated Periplasmic Glucans (OPGs), whose concentration generally decreases with an increase in external osmolarity (Bohin, 2000). However, the role of this enzyme has not been fully elucidated and no data on *Nitrosomonas*’s OPGs is currently available (Hanoulle et al., 2004).

Ultimately, the analysis of the proteomic data in relation to osmolytes confirmed that *N. europaea* modified several enzymes related to osmolytes production and most likely uses these molecules in response to a high salt concentration. Such a response, although beneficial for the overall cell physiology, is a nutrient- and energy-demanding process. Schimel et al. (2007) reported that the production of osmolytes, can potentially add up to 30–40% of the total carbon and 60% of the total nitrogen demands, resulting in a 2–3-fold increase in energetic cost for the bacteria (Schimel et al., 2007). Further quantification of the actual metabolic costs and their effect on bioprocess efficiency, can help the optimization process of nitrification in saline environments.

### Energy Production Enzymes of *N. europaea* at 30 mS cm^-1^: The Oxidative Pentose Phosphate Pathway Nexus

The results of the activity tests, which indicated no major modification in the ammonia oxidation rate at 30 mS cm^-1^, was not contradicted by the proteomic results, as no consistent variation in the ammonia oxidation apparatus was detected. Proteomic analysis did not identify the proteins involved in nitrite oxidation, therefore further investigations are necessary.

These results seem to indicate that no energy surplus can be provided for the maintenance of the stress response mechanisms. However, major alterations in carbon metabolism (Supplementary Table S4 and Figure S4) were observed, starting from an increase of carbonic anhydrase, a fundamental enzyme for the CO_2_ fixation via the Calvin–Benson–Bassham (CBB) process (Supplementary Figure S4A), utilized by *N. europaea* for carbon assimilation (Wei et al., 2004), potentially to be utilized for organic osmolytes production. A similar increase was observed in the transcriptome of *N. europaea* subjected to inorganic carbon limitations and was linked to a higher need for intracellular carbon (Mellbye et al., 2016).

The analysis of the carbon metabolism of *N. europaea* indicates a higher abundance of the enzymes involved in the oxidative phase of the PPP (Supplementary Figure S4B), responsible for NADPH production that can potentially be used to cope with the demands of the stress response mechanisms. Additionally, the enzyme responsible for the conversion of xylulose-5P into glyceraldehyde-3P, was more abundant at 30 mS cm^-1^. Alongside a halved abundance of the committing enzymes of terpene biosynthesis, it could contribute to the accumulation of glyceraldehyde-3P. Notably, glyceraldehyde-3P is the first intermediate of the pay-off phase of glycolysis, that generates reducing power (NADH) and ATP. It is known that *N. europaea* presents all the genes for glycolysis and the Krebs cycle (Chain et al., 2003; Schmidt, 2009) and, although not presenting significant differences among conditions, all these enzymes were detected (Supplementary Figures S4C,D). Whether or not the accumulation of glyceraldehyde-3P can help the cells in producing energy, represents an interesting challenge for future biochemical research.

## Conclusion

For the first time an integrated description of salt-induced metabolic and molecular shifts was reported, contributing to our understanding of the impact of the salinity on the nitrifiers’ physiology.

In contrast with previous reports, ammonia oxidation by *Nitrosomonas* spp. was not found to be more sensitive than nitrite oxidation to salinity. On the contrary, *N. europaea* and *N. ureae* activity were not perturbed by salinity of up to 30 mS cm^-1^, while the nitrite oxidation rate by *N. winogradskyi* significantly decreased to 42% of residual activity, compared to its activity at 5 mS cm^-1^. In the case of *N. vulgaris*, the presence of AOB and/or ammonia toxicity likely reduced activity. The combination of the proteomic analysis and the activity tests indicated that nitrogen metabolism of *N. europaea* was not primarily affected by salinity, while its carbon metabolism was subject to more sensitive changes toward stress. Higher abundance of enzymes involved in NADPH production through the PPP, confirm the expression of pathways identified at the genomic level by Chain et al. (2003). Additionally, response mechanisms to high salinity, commonly described in other microorganisms, were confirmed in *N. europaea* (osmolytes production, regulation of cell turgor and an oxidative stress response) and *N. winogradskyi* (an oxidative stress response).

The use of pure cultures and synthetic microbial communities proved to be a useful and flexible tool for an in-depth analysis of the microbial response. While our study focused on the proteomic response to salinity, previous work has utilized pure cultures and synthetic communities to validate genome-scale models of nitrification (Mellbye et al., 2018) and develop metabolic network models of wastewater treatment (Perez-Garcia et al., 2014). Implementation of such a community with bacteria, with different metabolic functions, could lead to the development of the modeling of and predictive tools for natural and engineered systems (Perez-Garcia et al., 2016). The addition of ureolytic heterotrophs for example, is crucial for urine nitrification bioprocesses, in which urea need to be hydrolyzed into ammonium, to be nitrified and have the organic compounds removed. The use of representative strains of different environments could instead provide insights on microbial interactions and differences among ecosystems, linking axenic and environmental studies.

## Author Contributions

CI was responsible for executing the activity tests, processing the proteomic data, and drafting the manuscript. BL and RW were responsible for the proteomic analysis. SV, NB, and PC designed the research and supervised the entire study. All authors contributed to the editing and correction of the manuscript.

## Conflict of Interest Statement

The authors declare that the research was conducted in the absence of any commercial or financial relationships that could be construed as a potential conflict of interest.
